# Work expectations, their fulfillment, and exhaustion among radiologists of all career levels: what can be learned from the example of Germany

**DOI:** 10.1007/s00330-023-09510-6

**Published:** 2023-03-10

**Authors:** Isabel Molwitz, Christoph Kemper, Katharina Stahlmann, Thekla Helene Oechtering, Malte Maria Sieren, Saif Afat, Mirjam Gerwing, Andreas Michael Bucher, Corinna Storz, Marcel C. Langenbach, Martin Reim, Joachim Lotz, Vera Zagrosek-Regitz, Elif Can, Daniel Köhler, Jin Yamamura, Gerhard Adam, Bernd Hamm, Sarah Keller

**Affiliations:** 1 Department of Diagnostic and Interventional Radiology and Nuclear Medicine, University Medical Center Hamburg-Eppendorf, Hamburg, Germany; 2 Department of Radiology, Charité Universitaetsmedizin Berlin, corporate member of Freie Universität Berlin, Humboldt-Universität zu Berlin, and Berlin Institute of Health, Berlin, Germany; 3 Institute of Medical Biometry and Epidemiology, University Medical Center Hamburg-Eppendorf, Hamburg, Germany; 4 Department of Radiology and Nuclear Medicine, University Hospital Schleswig-Holstein Campus Lübeck, Lübeck, Germany; 5 Department of Radiology, University of Wisconsin, Madison, WI USA; 6 Department of Diagnostic and Interventional Radiology, Eberhard-Karls University, Tübingen, Germany; 7 Clinic of Radiology, Medical Faculty, University of Münster, Münster, Germany; 8 Institute for Diagnostic and Interventional Radiology, Goethe University Frankfurt, Frankfurt Am Main, Germany; 9 Department of Neuroradiology, Medical Center, Faculty of Medicine, University of Freiburg, Freiburg, Germany; 10 Institute for Diagnostic and Interventional Radiology, University Hospital Cologne, Cologne, Germany; 11 Cardiovascular Imaging Research Center, Department of Radiology, Massachusetts General Hospital, Harvard Medical School, Boston, MA USA; 12 Department of Radiology and Interventional Radiology, Tartu University Hospital, University of Tartu, Tartu, Estonia; 13 Institute for Diagnostic and Interventional Radiology, University Medical Center Göttingen, Göttingen, Germany; 14 Institute for Gender in Medicine, Charité Universitaetsmedizin Berlin, corporate member of Freie Universität Berlin, Humboldt-Universität zu Berlin, and Berlin Institute of Health, Berlin, Germany; 15 evidia Group, Berlin, Germany; 16 Department of Cardiology, University Hospital Zürich, University of Zürich, Zürich, Switzerland

**Keywords:** Job satisfaction, Burnout, Empowerment, Internship and residency, Radiologists

## Abstract

**Objectives:**

To evaluate work expectations of radiologists at different career levels, their fulfillment, prevalence of exhaustion, and exhaustion-associated factors.

**Methods:**

A standardized digital questionnaire was distributed internationally to radiologists of all career levels in the hospital and in ambulatory care via radiological societies and sent manually to 4500 radiologists of the largest German hospitals between December 2020 and April 2021. Statistics were based on age- and gender-adjusted regression analyses of respondents working in Germany (510 out of 594 total respondents).

**Results:**

The most frequent expectations were “joy at work” (97%) and a “good working atmosphere” (97%), which were considered fulfilled by at least 78%. The expectation of a “structured residency within the regular time interval” (79%) was more frequently judged fulfilled by senior physicians (83%, odds ratio (OR) 4.31 [95% confidence interval (95% CI) 1.95–9.52]), chief physicians (85%, 6.81 [95% CI 1.91–24.29]), and radiologists outside the hospital (88%, 7.59 [95% CI 2.40–24.03]) than by residents (68%). Exhaustion was most common among residents (physical exhaustion: 38%; emotional exhaustion: 36%), in-hospital specialists (29%; 38%), and senior physicians (30%; 29%). In contrast to paid extra hours, unpaid extra hours were associated with physical exhaustion (5–10 extra hours: OR 2.54 [95% CI 1.54–4.19]). Fewer opportunities to shape the work environment were related to a higher probability of physical (2.03 [95% CI 1.32–3.13]) and emotional (2.15 [95% CI 1.39–3.33]) exhaustion.

**Conclusions:**

While most radiologists enjoy their work, residents wish for more training structure. Ensuring payment of extra hours and employee empowerment may help preventing burnout in high-risk groups.

**Key Points:**

• *Most important work expectations of radiologists who work in Germany are “joy at work,” a “good working atmosphere,” “support for further qualification,” and a “structured residency within the regular time interval,” with the latter containing potential for improvement according to residents.*

• *Physical and emotional exhaustion are common at all career levels except for chief physicians and for radiologists who work outside the hospital in ambulatory care.*

• *Exhaustion as a major burnout criterion is associated with unpaid extra hours and reduced opportunities to shape the work environment.*

**Supplementary Information:**

The online version contains supplementary material available at 10.1007/s00330-023-09510-6.

## Introduction

For over 20 years, staff shortage in radiology has been discussed [1], a problem aggravated by the augmented workload in diagnostic and interventional radiology and the increasing number of radiologists working part-time. With respect to the workload, a growth of 70% relative value units per full-time radiologist has been described for the United States of America (US) between 1991–1992 and 2006–2007 [2], a trend which continues as exemplified by a large European hospital, reporting an increase of CT studies, e.g., for trauma, pulmonary embolism, or aortic dissection of 500% between 2006 and 2020 [3]. Simultaneously, burnout, being defined as emotional exhaustion, detachment from the job or depersonalization, and a sense of lack of accomplishment as a response to continued work-related stress [4], is increasingly common among radiologists. In a multicenter US study across 24 different medical disciplines, after correction for age, gender, relationship status, and working time, radiology was one of five specialties with a significantly higher risk of burnout [5]. In that study, 61% of 261 radiologists reported symptoms of burnout. Burnout occurs among all career steps. High rates of emotional exhaustion were already found in radiological trainees with 50–53% [6, 7]. Over the last decade, the prevalence of burnout symptoms among radiologists has been found to further increase [8]. There is thus a discrepancy between the workload and available human resources with dissatisfied radiologists tending to reduce their working hours, change the employer, move abroad, or even give up clinical practice [9].

It is therefore vital for hospital managements, department heads, and radiological practices to effectively recruit students and young colleagues for a radiological residency and to hold qualified radiologists by ensuring their motivation and well-being. To this end, knowledge on radiologists’ work expectations, on their fulfillment, on the prevalence of exhaustion as a major burnout criterion, and on factors associated with exhaustion is necessary. Interestingly, while it has already been reported for final-year students that interdisciplinary work and good working conditions are main motivations to pursue a career in radiology [10], there are only a few studies which have at least in part assessed work conditions and their satisfaction among radiologists. One survey about the quality of the radiological residency in Germany found 13% of residents to be dissatisfied with their work conditions and identified workload, insufficient training, and lack of supervision as contributing factors [9]. In interventional radiology training, mentoring and structured feedback have been described as positively correlated with work satisfaction [11].

Therefore, the aim of this survey was to investigate work expectations of radiologists at different career levels in the hospital and in ambulatory care and to evaluate how well these expectations are fulfilled. Furthermore, we aimed to determine the prevalence of exhaustion and to identify factors that may contribute to exhaustion.

## Methods

### Data collection

For this survey, an institutional review board exemption (Charité – University Medicine Berlin, EA1/174/20) was obtained. All analyses were conducted in compliance with the revised Declaration of Helsinki.

A questionnaire was distributed via the German Roentgen Society’s (DRG) conference of university professors (KLR) and German Young Radiology Forum, the European Society of Radiology (ESR) and its Radiology Trainee Forum, and the Radiological Society of North America’s (RSNA) Resident and Fellow Committee and manually sent to 4500 radiologists of the biggest German hospitals between December 2020 and April 2021. It consisted of 66 items about (a) professional background, (b) current professional situation, (c) job satisfaction, (d) career aims, and (e) personal information. To enable quantitative analyses of the participants’ responses, besides open questions, Likert scales, e.g., to assess the agreement to different work expectations, were employed. The complete questionnaire is provided as Supplement 1 to this article.

As most respondents worked in Germany and the number of participants from other countries was not representative, this article only employs data from participants with German affiliations.

### Statistics

Continuous variables are provided as mean and standard deviation (SD), and categorical variables as absolute and relative frequencies.

All analyses were adjusted for age and gender. Linear regression models were employed for continuous variables (e.g., number of children), multinomial logistic regressions for multi-categorical variables (e.g., current position), and binary logistic regressions for binary variables (e.g., part-time). Independent binary logistic regressions were used for the association of socioeconomic aspects and work conditions with job expectations, their fulfillment, exhaustion, and satisfaction with support systems. Likert scale responses were dichotomized into, e.g., very important/important vs. not so important/not important, or always/mainly fulfilled vs. hardly/not fulfilled. To increase group size and thus statistical reliability, for comparisons between participants at different career levels, the categories senior physicians and leading senior physicians from the questionnaire were combined. For employed and self-employed radiologists in ambulatory care, separate and combined analyses were conducted.

Concerning missing variables, participants who did not indicate their gender (*n* = 6) were excluded from the analyses as were participants of diverse gender due to small numbers (*n* = 2). Radiologists working in other countries than Germany were few (*n* = 84 vs. *n* = 510), originated from 33 different countries, and were thus excluded to ensure validity and comparability of the analyses.

Because of the explorative study design, *p*-values were not adjusted for multiplicity and should be considered as descriptive summary measures. All calculations were performed in SAS 9.4 (SAS Institute).

## Results

### Study collective

The final analyzed sample consisted of 510 radiologists and radiological residents with German affiliations. Of these, 237 (47%) were female (42 years ± 10 SD) and 273 (54%) were male (47 years ± 11 SD, *p*-value < 0.001).

Most participants were senior physicians or leading senior physicians (*n* = 168, 33%), followed by residents (*n* = 146, 29%), radiologists, who worked outside the hospital as employed (*n* = 28) or self-employed (*n* = 45) radiologists in ambulatory care, e.g., in radiological practices (together *n* = 73, 14%), chief physicians or clinical directors (*n* = 64, 13%), and in-hospital specialists (*n* = 56, 11%). Gender was equally distributed, except for chief physicians, who were ten times more likely to be male (odds ratio (OR) 10.72 [95% confidence interval (95% CI) 3.63–31.64]) and self-employed radiologists in ambulatory care who were four times more likely to be male (OR 4.10 [95% CI 1.57–10.71]).

Most participants were interested in diagnostic radiology (*n* = 362, 71%), followed by interventional radiology (*n* = 193, 38%), neuroradiology (*n* = 171, 34%), pediatric radiology (*n* = 53, 10%), and nuclear medicine (*n* = 28, 5%). Men were more likely to be interested in interventional radiology (OR 3.00 [95% CI 2.02–4.44]) and less interested in pediatric radiology (OR 0.43 [95% CI 0.23–0.79]) than women. There was no relevant gender discrepancy concerning the interest in neuroradiology (OR 1.44 [95% CI 0.98–2.11]).

The average number of children per participant was 1.3 ± 1.2 SD. Of male participants with children, 31% had taken a parental leave (6 months ± 8 SD) and of female participants with children 83% (21 months ± 12 SD). Participants in part-time (men: 7%, women: 41%) worked an average of 29 hours ± 7 SD. Most residents had fixed-term contracts (94%), while at all other career levels permanent contracts were most common. After correction for career level, there were no significant differences in contract duration between both genders.

For further details about the study population, please see Table 1.

**Table 1:** Characteristics of the study population. Significant differences between female and male participants are italicized

	**Female**	**Male**	**Total**	**Difference**^**a**^** (Ref: female)**
***N* (%)**	237 (46.5)	273 (53.5)	510 (100)	
	**Mean (SD)**			***β* (*p*-value)**
**Age (years)**	41.6 (10.1)	46.8 (11.1)	44.4 (11.0)	5.18 (< .001)
**Children per participant**	1.0 (1.1)	1.5 (1.3)	1.3 (1.2)	0.19 (0.05)
*Children per participant (if children* = *yes)*	*2.0 (0.7)*	*2.2 (0.86)*	*2.2 (0.83)*	*0.24 (0.02)*
*Parental leave in months (if children* = *yes)*	*20.5 (12.1)*	*6.2 (8.3)*	*15.4 (12.8)*	* − 14.21 (*< *.001)*
**Children**	***N* (%)**			**Odds ratio (95% CI)**
Yes	123 (51.9)	179 (65.6)	302 (59.2)	1.11 (0.73, 1.69)
No	114 (48.1)	94 (34.4)	208 (40.8)	Ref
**Parental leave** (if children = yes)				
*Yes*	*102 (82.9)*	*56 (31.3)*	*158 (52.3)*	*0.12 (0.06, 0.21)*
No	21 (17.1)	123 (68.7)	144 (47.7)	Ref
**Current position**				
Resident	87 (36.9)	59 (21.8)	146 (28.8)	Ref
Specialist	38 (16.1)	18 (6.6)	56 (11.1)	0.79 (0.35, 1.77)
(Leading) senior physician	76 (32.2)	92 (34.0)	168 (33.1)	1.84 (0.90, 3.76)
*Employed or self-employed in outpatient care*	*28 (11.9)*	*45 (16.6)*	*73 (14.4)*	*2.31 (1.01, 5.29)*
*Chief physician*	*7 (3.0)*	*57 (21.0)*	*64 (12.6)*	*10.72 (3.63, 31.64)*
**Did your residency took longer than intended?**				
*Yes*	*116 (52.3)*	*114 (43.2)*	*230 (47.3)*	*0.67 (0.47, 0.98)*
No	106 (47.8)	150 (56.8)	256 (52.7)	Ref
**Working time**				
Full-time	137 (59.1)	250 (92.6)	387 (77.1)	Ref
*Part-time*	*95 (41.0)*	*20 (7.4)*	*115 (22.9)*	*0.09 (0.05, 0.16)*
**Working time (if children = yes)**				
Full-time	43 (35.2)	163 (92.1)	206 (68.2)	Ref
*Part-time*	*79 (64.8)*	*14 (7.9)*	*93 (30.8)*	*0.05 (0.03, 0.11)*
**Contract duration**				
< 1 year	12 (5.1)	8 (3.0)	20 (4.0)	0.72 (0.27, 1.91)
1–3 years	54 (22.9)	39 (14.7)	93 (18.5)	0.81 (0.45, 1.46)
> 3 years	34 (14.4)	23 (8.7)	57 (11.4)	0.79 (0.39, 1.62)
Permanent	136 (57.6)	196 (73.7)	332 (66.1)	Ref
**Interest**				
Diagnostic radiology	166 (70.1)	196 (71.8)	362 (71.0)	-
*Interventional radiology*	*62 (26.2)*	*131 (48.0)*	*193 (37.8)*	*3.00 (2.02, 4.44)*
Neuroradiology	70 (29.5)	101 (37.0)	171 (33.5)	1.44 (0.98, 2.11)
*Pediatric radiology*	*34 (14.4)*	*19 (7.0)*	*53 (10.4)*	*0.43 (0.23, 0.79)*
Nuclear medicine	9 (3.8)	19 (7.0)	28 (5.5)	1.74 (0.75, 4.04)

Abbreviations: Ref reference, *SD* standard deviation, *95% CI* 95% confidence interval. ^a^All regression models included gender as independent variable and were adjusted for age

### Work expectations

**Fig. 1:**
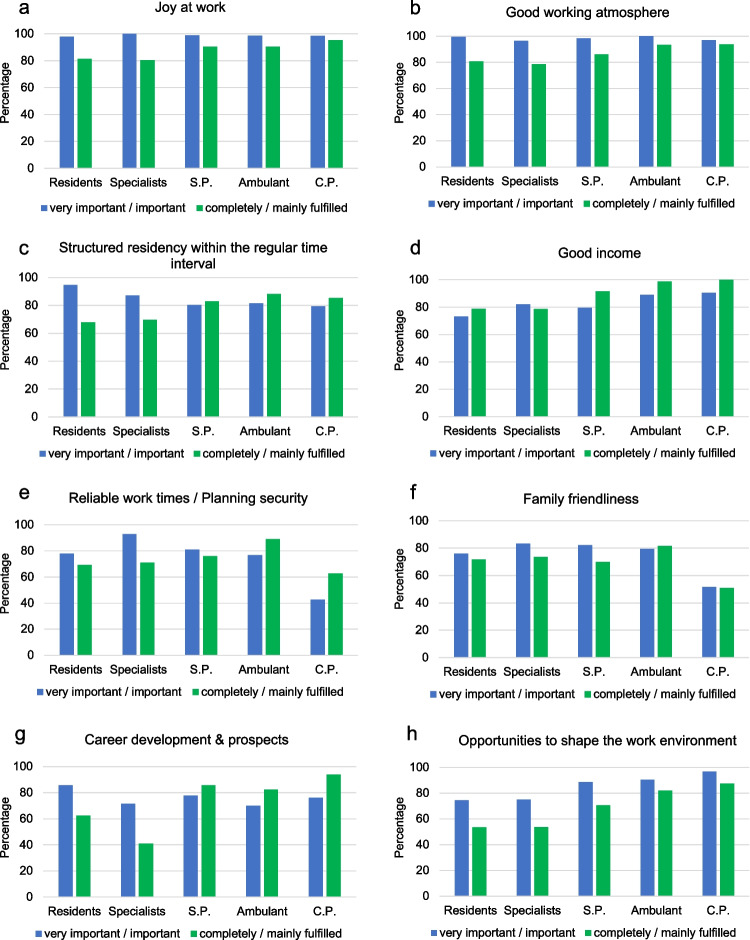
**a**–**h** Work expectations and their fulfillment. Participants (%) who considered the respective expectation as very important or important (blue) and as completely or mainly fulfilled (green); S.P. senior physicians, C.P. chief physicians. The most important expectations “joy at work” and a “good working atmosphere” were relatively well fulfilled. The expectation of residents concerning a “structured residency within the regular time interval” could be better met. Expectations of a good income were more than met. Family friendliness was best fulfilled among radiologists in ambulatory care. Career prospects and opportunities to shape the work environment were better fulfilled at higher career levels

The work expectations most frequently rated as important with at least 96% by the participants of each career level were “joy at work” and a “good working atmosphere” (Fig. 1A, B). Thirdly, participants of all career levels judged “support for further qualification” as relevant (≥ 84%). More than 79% considered a “structured residency within the regular time interval” as important, which refers to an organized curriculum with rotations to all modalities and sections within the department to qualify for the board exam within the standard required time of residency (in Germany 60 months). Highest rates concerning this expectation were found among residents (95%) (Fig. 1C). Compared to the residents, senior physicians considered this aspect less important (OR 0.29 [95% CI 0.11–0.76]).

In reference to the residents (73%), a “good income” was more frequently judged important by senior physicians (80%, OR 2.18 [95% CI 1.05–4.51]), by chief physicians (91%, OR 6.69 [95% CI 2.03–22.03]), and by both employed (OR 5.07 [95% CI 1.27–20.20]) and self-employed (OR 5.11 [95% CI 1.56–16.71]) radiologists in ambulatory care (together: 89%) (Fig. 1D). The only group which judged “reliable working time and planning security” more frequently important than residents were specialists (OR 4.30 [95% CI 1.38–13.38]) (Fig. 1E). Also, “family friendliness” was most important for specialists (83%). It was the least important for chief physicians (52%) (Fig. 1F), and slightly less important for men (70%, OR 0.52 [95% CI 0.34–0.81]) than for women (82%).

Concerning “opportunities to shape the work environment,” relevant differences were found between residents (74%) and chief physicians (97%, OR 5.41 [95% CI 1.00–29.43]) (Fig. 1H). On the contrary, while “career development and prospects” were important for all groups, highest rates of agreement with 86% were found among residents (Fig. 1G).

### Fulfillment of work expectations

The only one of the above-mentioned expectations that was more frequently considered fulfilled than judged important among all groups except for specialists was a good income (Fig. 1D).

Both expectations “joy at work” and a “good working atmosphere” which were most frequently named as important were judged as mainly or completely fulfilled by at least 79% at all career levels (Table 2). With the residents as reference, “joy at work” was more likely to be considered fulfilled among senior physicians (OR 2.75 [95% CI 1.12–6.75]) and chief physicians (OR 6.19 [95% CI 1.34–28.68]). Also, a “good working atmosphere” was more likely judged fulfilled by senior physicians (OR 3.04 [95% CI 1.26–7.33]), chief physicians (OR 7.07 [95% CI 1.72–29.06]), and radiologists in ambulatory care (OR 6.83 [95% CI 2.00–23.38]). “Support for further qualification” was the least fulfilled for residents (64%), with senior physicians (OR 2.47 [95% CI 1.23–4.94]), chief physicians (OR 5.12 [95% CI 1.65–15.90]), and radiologists in ambulatory care (OR 7.56 [95% CI 2.61–21.85]) being more likely to consider their expectations met.

**Table 2:** Physical exhaustion among radiologists. Significant differences between the reference group and respective group of interest are italicized

Physical exhaustion	Never	Seldom	Sometimes	Often	Always	Total	Difference^a^ (Ref: never/seldom)
	***N* (%)**	***N* (%)**	***N* (%)**	***N* (%)**	***N* (%)**	***N* (%)**	**OR (95% CI)**
**Gender**							*N* = 507
Female	7 (3.0)	64 (27.0)	85 (35.9)	75 (31.7)	6 (2.5)	237 (46.5)	Ref
*Male*	*15 (5.5)*	*104 (38.1)*	*88 (32.2)*	*60 (22.0)*	*6 (2.2)*	*273 (53.5)*	*0.59 (0.41, 0.86)*
**Current position**							*N* = 504
Resident	8 (5.5)	38 (26.0)	45 (30.8)	49 (33.6)	6 (4.1)	146 (28.8)	Ref
Specialist	2 (3.6)	16 (28.6)	22 (39.3)	15 (26.8)	1 (1.8)	56 (11.1)	1.00 (0.49, 2.02)
(Leading) senior physician	4 (2.4)	54 (32.1)	60 (35.7)	46 (27.4)	4 (2.4)	168 (33.1)	1.03 (0.56, 1.88)
Employed or self-employed in outpatient care	4 (5.5)	34 (46.6)	23 (31.5)	11 (15.1)	1 (1.4)	73 (14.4)	0.53 (0.26, 1.09)
Chief physician	3 (4.7)	26 (40.6)	22 (34.4)	13 (20.3)	0 (0.0)	64 (12.6)	0.80 (0.35, 1.87)
**Paid overtime per week**							*N* = 506
None	10 (4.3)	81 (34.8)	76 (32.6)	59 (25.3)	7 (3.0)	233 (45.8)	Ref
1–4 h	9 (7.1)	43 (33.9)	46 (36.2)	29 (22.8)	0 (0.0)	127 (25.0)	0.72 (0.45, 1.16)
5–10 h	2 (1.6)	34 (26.4)	45 (34.9)	43 (33.3)	5 (3.9)	129 (25.3)	1.49 (0.93, 2.41)
> 10 h	1 (5.0)	10 (50.0)	5 (25.0)	4 (20.0)	0 (0.0)	20 (3.9)	0.51 (0.20, 1.31)
**Unpaid overtime per week**							*N* = 506
None	13 (5.4)	96 (39.7)	75 (31.0)	56 (23.1)	2 (0.8)	242 (47.5)	Ref
1–4 h	4 (3.6)	33 (29.7)	38 (34.2)	32 (28.8)	4 (3.6)	111 (21.8)	1.41 (0.87, 2.28)
*5–10 h*	*5 (3.4)*	*24 (20.9)*	*47 (40.9)*	*34 (29.6)*	*5 (4.4)*	*115 (22.6)*	*2.54 (1.54, 4.19)*
> 10 h	0 (0.0)	15 (36.6)	12 (29.3)	13 (31.7)	1 (2.4)	41 (8.1)	1.96 (0.95, 4.04)
**Opportunities to shape the work environment**							*N* = 491
Complete/mainly fulfilled	19 (5.7)	127 (37.8)	118 (35.1)	68 (20.2)	4 (1.2)	336 (68.0)	Ref
*Hardly/not fulfilled*	*3 (1.9)*	*36 (22.8)*	*49 (31.0)*	*63 (39.9)*	*11 (5.7)*	*158 (32.0)*	*2.03 (1.32, 3.13)*
**Total**	22 (4.3)	168 (32.9)	173 (33.9)	135 (26.5)	12 (2.4)	510 (100.0)	

Abbreviations: *Ref* reference, *OR* odd ratio, *95% CI* 95% confidence interval. ^a^All regressions were adjusted for gender and age

Concerning a “structured residency within the regular time,” senior physicians (83%, OR 4.31 [95% CI 1.95–9.52]), chief physicians (85%, OR 6.81 [95% CI 1.91–24.29]), and radiologists in ambulatory care (88%, OR 7.59 [95% CI 2.40–24.03]) were more likely to report this item as fulfilled than residents (68%).

According to 52% of the female and 43% of the male participants, their residency took longer than intended (male vs. female OR 0.67 [95% CI 0.47–0.98]) (Table 1). Reasons for a prolonged residency were change of employer (17%), parental leave (14%), part-time (10%), missing availability of rotation positions (10%), research (9%), or a stay abroad (4%).

Compared to the residents, “career development and prospects” were more frequently fulfilled for senior physicians (OR 7.56 [95% CI 3.37–16.94]), chief physicians (OR 23.80 [95% CI 5.42–104.54]), and radiologists in ambulatory care (OR 6.40 [95% CI 2.43–16.86]).

Fulfillment of “reliable working time and planning security” exceeded expectations among chief physicians and radiologists in ambulatory care (Fig. 1E). Radiologists in ambulatory care were also the only ones who were significantly more likely than residents to consider their expectations about “family friendliness” (82%, OR 2.61 [95% CI 1.09–6.27]) fulfilled. Furthermore, compared to residents “reliable working time and planning security” were more often fulfilled in ambulatory care with a higher probability among employed (OR 5.72 [95% CI 1.21–27.11]) than among self-employed (OR 2.83 [95% CI 0.95–8.38]) radiologists. Across all career levels, there was no difference in fulfillment of family friendliness according to men (70%) and women (71%) (OR 1.09 [95% CI 0.72–1.64]).

### Physical exhaustion

**Fig. 2:**
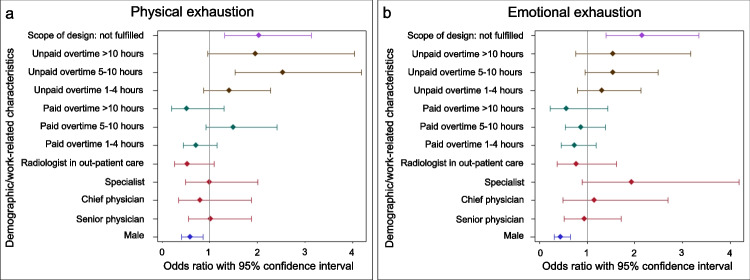
Forest plot of the association of demographics and work-related characteristics with physical (**a**) and emotional (**b**) exhaustion. Unpaid extra hours and missing opportunities to shape the work environment (scope of design) were associated with physical and emotional exhaustion. Physical and emotional exhaustion were less likely among men than among women. Fulfilled opportunities to shape the work environment, no unpaid extra hours, no paid extra hours, residents, and female participants served as group of reference and are thus not displayed

Feeling always or often physically drained was most common among residents (38%), followed by senior physicians (30%), and specialists (29%). Rates were lowest among chief physicians (20%) and radiologists in ambulatory care (16%). While the differences between career levels were not statistically relevant (Table 2), the probability for physical exhaustion among men (OR 0.59 [95% CI 0.41–0.56]) was less pronounced than among women (Fig. 2A). About a quarter of all participants (28%) reported to attend work even when feeling ill, with men being less likely to do so (OR 0.65 [95% CI 0.44–0.95]).

Across all participants, working unpaid extra hours was associated with physical exhaustion (5–10 extra hours: OR 2.54 [95% CI 1.54–4.19], > 10 extra hours: 1.96 [95% CI 0.95–4.04]) (Fig. 2A). Similarly, reduced opportunities to shape the work environment were associated with physical exhaustion (OR 2.03 [95% CI 1.32–3.13]) (Fig. 2A). Paid extra hours, paid or unpaid on-call service, or presence service did not show any association with physical exhaustion (Table 2).

### Emotional exhaustion

The probability to feel always or often emotionally exhausted was highest among specialists (38%) and residents (36%), followed by senior physicians (29%). Again, it was lowest for chief physicians (20%) and radiologists in ambulatory care (21%). Differences were not statistically relevant between the career levels, but between men (OR 0.45 [95% CI 0.31–0.66]) and women.

Reduced opportunities to shape the work environment were associated with emotional exhaustion (OR 2.15 [95% CI 1.39–3.33]). While there was a tendency for unpaid extra hours to be related to emotional exhaustion (Fig. 2B), this was not statistically relevant (Table 3).

**Table 3:** Emotional exhaustion among radiologists. Significant differences between the reference group and respective group of interest are italicized

Emotional exhaustion	Never	Seldom	Sometimes	Often	Always	Total	Difference^a^ (Ref: never/seldom)
	***N* (%)**	***N* (%)**	***N* (%)**	***N* (%)**	***N* (%)**	***N* (%)**	**OR (95% CI)**
**Gender**							*N* = 507
Female	9 (3.8)	56 (23.6)	78 (32.9)	85 (35.9)	9 (3.9)	237 (46.5)	Ref
*Male*	*22 (8.1)*	*105 (38.5)*	*88 (32.2)*	*52 (19.1)*	*6 (2.2)*	*273 (53.5)*	*0.45 (0.31, 0.66)*
**Current position**							*N* = 504
Resident	11 (7.5)	39 (26.7)	43 (29.5)	46 (31.5)	7 (4.8)	146 (28.8)	Ref
Specialist	3 (5.4)	9 (16.1)	23 (41.1)	20 (35.7)	1 (1.8)	56 (11.1)	1.94 (0.90, 4.18)
(Leading) senior physician	9 (5.4)	58 (34.5)	52 (31.0)	43 (25.6)	6 (3.6)	168 (33.1)	0.94 (0.52, 1.71)
Employed or self-employed in outpatient care	6 (8.2)	28 (38.4)	24 (32.9)	15 (20.6)	0 (0.0)	73 (14.4)	0.78 (0.37, 1.61)
Chief physician	2 (3.1)	25 (39.1)	24 (37.5)	12 (18.8)	1 (1.6)	64 (12.6)	1.15 (0.49, 2.69)
**Paid overtime per week**							*N* = 506
None	11 (4.7)	74 (31.8)	83 (35.6)	59 (25.3)	6 (2.6)	233 (45.8)	Ref
1–4 h	12 (9.5)	36 (28.4)	42 (33.1)	32 (25.2)	5 (3.9)	127 (25.0)	0.73 (0.45, 1.19)
5–10 h	5 (3.9)	43 (33.3)	37 (28.7)	40 (31.0)	4 (3.1)	129 (25.3)	0.87 (0.55, 1.38)
> 10 h	3 (15.0)	7 (35.0)	4 (20.0)	6 (30.0)	0 (0.0)	20 (3.9)	0.56 (0.22, 1.43)
**Unpaid overtime per week**							*N* = 506
None	19 (7.9)	83 (34.3)	79 (32.6)	58 (24.0)	3 (1.2)	242 (47.5)	Ref
1–4 h	5 (4.5)	31 (27.9)	40 (36.0)	29 (26.1)	6 (5.4)	111 (21.8)	1.31 (0.80, 2.13)
5–10 h	6 (5.2)	31 (27.0)	33 (28.7)	39 (33.9)	6 (5.2)	115 (22.6)	1.54 (0.96, 2.49)
> 10 h	1 (2.4)	15 (36.6)	14 (34.2)	11 (26.8)	0 (0.0)	41 (8.1)	1.55 (0.76, 3.16)
**Opportunities to shape the work environment**							*N* = 491
Complete/mainly fulfilled	26 (7.7)	124 (36.9)	115 (34.2)	66 (19.6)	5 (1.5)	336 (68.0)	Ref
*Hardly/not fulfilled*	*3 (1.9)*	*35 (22.2)*	*47 (29.8)*	*64 (40.5)*	*9 (5.7)*	*158 (32.0)*	*2.15 (1.39, 3.33)*
**Total**	31 (6.1)	161 (31.6)	166 (32.6)	137 (26.9)	15 (2.9)	510 (100.0)	

Abbreviations: *Ref* reference, *OR* odd ratio, *95% CI* 95% confidence interval. ^a^All regressions were adjusted for gender and age

Of all respondents, 24% regularly struggled to relax after work. About 10% felt frequently burdened by problematic decisions in patient care, with a smaller probability among male participants (OR 0.50 [95% CI 0.34–0.72]).

### Support systems

Of all participants, 86% considered their work always or mostly meaningful. About half of them (54%) were satisfied or rather satisfied with interviews with their supervisors for career development, while 46% were not. Men were more likely than women to be satisfied with such interviews (OR 1.86 [95% CI 1.25–2.78]), as were senior physicians compared to residents (OR 2.45 [95% CI 1.28–4.68]). About half of the participants (52%) were satisfied or rather satisfied with supervision of their work.

Men (OR 1.62 [95% CI 1.09–2.41]) and senior physicians (OR 1.91 [95% CI 1.01–3.62]) were more likely to be satisfied with support in case of critical events than women or residents, respectively.

The most important source of support among all participants were their partners (86%), followed by family (84%), friends (70%), colleagues (70%), and supervisors (50%).

## Discussion

This study surveyed work expectations, their fulfillment, and exhaustion among 510 radiologists of all career levels with German affiliations working in hospitals and ambulatory care. The most frequent expectations joy at work and a good working atmosphere were both considered mainly fulfilled among all participants. The expectations of residents concerning a structured residency within the regular time interval could still be better met. Both chief physicians and radiologists in ambulatory care were most satisfied with reliability of their working hours and planning security, while family friendliness was best fulfilled among radiologists in ambulatory care.

Physical and emotional exhaustion were common among all groups except for chief physicians and radiologists in ambulatory care. Exhaustion was associated with unpaid extra hours and reduced opportunities to shape the work environment.

This study’s results demonstrate that soft criteria such as joy at work and a good working atmosphere are even more demanded than hard facts like a good income among radiologists with German affiliations. This is of interest, because while there are many international studies on burnout in radiology, in a literature search no studies could be identified, which specifically assessed joy at work among radiologists from other countries. However, joy at work increases coping mechanisms to work-related stressors [12]. Furthermore, happiness and job satisfaction influence work performance [13, 14]. Therefore, this study’s results can serve as an inspiration to consider and evaluate joy at work as a potential major motivation for radiologists in other countries, as well.

Also, the importance of family friendliness was judged high by the large majority in all groups apart from chief physicians (52%), but these expectations were not yet sufficiently fulfilled for radiologists who work in hospitals. This is relevant, because family friendliness is an internationally increasing expectation of the generation Z, as demonstrated, e.g., by a study among students in Poland, which found “family, health, and friendship” to be major values for 60%, while “professional carrier development and high salary” were important for only 11% [15]. Moreover, family friendliness is likely of increasing international importance because of the increasing number of women in medicine and radiology worldwide [16, 17]. Thus, the association of work-to-family conflicts with job satisfaction [18], which causes low job performance and high turnover rates [19], will become an even more relevant problem. Supervisors could intervene as their support in balancing work and family issues can contribute to higher job satisfaction [14]. To support their employees, simple tools such as interviews for career development can be employed, which have been found to be associated with job satisfaction and lower psychosocial workload [9].

Concerning the identified potential to further improve the radiological residency’s structure and ensure timely board exam qualification, this study’s results are in accordance with prior findings. In 2018, the German Young Radiology Forum found a structured curriculum with a transparent rotation plan to the different modalities and sections within the department to be the second most effective training instrument after supervision with case discussions [9]. While it is specific for Germany that there are no monetary incentives for teaching of residents, the desire of structured training seems to be an issue for other European countries as well, as demonstrated by the existence of not only national but also the ESR’s European Training Curricula for the radiological residency [20, 21]. To achieve the learning objectives of such structured curricula, easily accessible online material would be helpful. Indeed, thanks to the catalyzation of digital teaching during the COVID-19 pandemic [22], there are options like the EDiR Preparatory Micro Courses or RADUCATION, a free-of-charge platform by the German Young Radiology Forum [23], on whose existence awareness could be increased.

Furthermore, this study has important implications concerning exhaustion as a burnout criterion. Percentages in this study (up to 38%) were lower than in several US studies which reported symptoms of burnout of greater than 50% among radiological residents [5–7]. This may be due to geographical differences in workload or support systems. However, with more than one-third of residents and specialists suffering from exhaustion, more than 16% of participants from all other groups feeling exhausted according to this study, and rates at least that high on an international level, exhaustion is a relevant threat to both the individual radiologist’s health and the overall work performance in radiology. Both aspects which were found to be associated with exhaustion: unpaid extra hours and reduced opportunities to shape the work environment, should thus be effectively addressed. Paying or reducing extra hours is likely also economically profitable, as long working times are known to negatively influence occupational health [24]. Concerning opportunities to shape the work environment, empowerment is crucial for job satisfaction [14], which again improves performance [13, 14, 19]. Radiologists should thus be given autonomy in their daily routine and be involved in decision-making processes.

Concerning female radiologists, among chief physicians and self-employed radiologists, there were significantly less women. This finding is not specific for Germany as a systematic review from 2021 which included 61 studies from all over the world (none from Germany) demonstrated [25]. Similarly, the lower interest of female participants in interventional radiology has been described before among female medical students from Europe to New Zealand [26]. Early exposure and direct pathways to interventional radiology, family friendliness, and information on radiation protection during pregnancy have been identified as potential approaches to increase female interest in interventional radiology [26].

While there was no relevant difference in the fulfillment of family friendliness between both genders, family friendliness was more important for women, who were also more likely to work part-time. Again, the higher amount of family care work [27] among women is in good agreement with other countries and likely also a major reason for why exhaustion was more common among female radiologists.

Limitations of this study are its observational design and its focus on Germany. Despite worldwide distribution of the questionnaire, most participants were working in Germany. Thus, statistical analyses were focused on these to avoid biased results. Some findings, such as soft criteria (e.g., “joy at work”) being more frequently rated as important than a good income, might be different in, e.g., Eastern European countries, where physicians’ salaries are lower. However, as far as comparable analyses have been performed, this study’s results are coherent to investigations from other countries. As discussed above, major results and conclusions are therefore likely transferable to radiologists on an international level. Finally, a participation bias, with respondents being more engaged or interested towards their work than non-respondents, may be existent. However, this study was advertised as a survey on radiological career aspects without any previous information on concrete questions. It is thus likely that people did not participate to report specifically on a certain aspect like work conditions or exhaustion.

In conclusion, this study provides insights into work expectations, their fulfillment, and exhaustion based on the example of radiologists with German affiliations. Joy at work and a good working atmosphere are major and mostly fulfilled expectations. There is potential for improvement concerning a structured residency within the regular time interval and family friendliness for in-hospital radiologists. Exhaustion is common especially among residents and specialists in the hospital and may be prevented by reducing unpaid extra hours and ensuring sufficient opportunities to shape the work environment.

## Supplementary Information

Below is the link to the electronic supplementary material.

Supplementary file1 (PDF 406 kb)

## Abbreviations

CT: Computed tomography
DRG: German Roentgen Society
ESR: European Society of Radiology
KLR: Conference of university professors
OR: Odds ratio
RSNA: Radiological Society of North America
SD: Standard deviation
US: United States

## Declarations

### Guarantor

The scientific guarantor of this publication is PD Dr. Sarah Keller.

### Statistics and biometry

One of the authors has significant statistical expertise. (Ms. Stahlmann, M.Sc. with expertise in medical biometry from the Institute of Medical Biometry and Epidemiology, University Medical Center Hamburg-Eppendorf, Germany, conducted all statistical analyses.)

### Methodology

• retrospective

• observational

• multicenter study (survey)
