# Supplementary material for: Work expectations, their fulfillment, and exhaustion among radiologists of all career levels: what can be learned from the example of Germany
Source: Eur Radiol. 2023 Mar 10;33(8):5664–74. doi: 10.1007/s00330-023-09510-6 (PMC9999063; doi:10.1007/s00330-023-09510-6)
Supplement: Supplementary file 1 — Supplementary file1 (PDF 406 kb) [file 330_2023_9510_MOESM1_ESM.pdf]

Factors determining the success for a scientific career

Dear study participant,

Thank you for taking the time to participate in our online questionnaire study. Answering the questionnaire will take about 15 minutes.

In this study we wish to determine which structural and personal factors are associated with a succesfull radiological career.

The questionnaire is divided into 5 parts:

1. Professional Background
2. Current professional Situation
3. Job Satisfaction and Personality Traits
4. Career Aims
5. Information about your Person and your private Circumstances

If you have any questions about the survey please do not hesitate to contact: i.molwitz@uke.de

There are 66 questions in this survey.

I. Information on Education and Professional Background

In which year did you complete your medical studies? *

Comment only when you choose an answer.

Please choose all that apply and provide a comment:

- [ ] Year (JJJJ):

- [ ] Not applicable

Have you possibly completed other courses of study? Please specify the degree and the field of study, if applicable. *

❗ Comment only when you choose an answer.

Please choose all that apply and provide a comment:

☐ No

☐ Master

☐ Bachelor

☐ Diploma

☐ No information

Other:

Have you completed a doctorate? *

❗ Choose one of the following answers
Please choose **only one** of the following:

- ◯ Yes
- ◯ No

What title did you gain through your promotion? *

Only answer this question if the following conditions are met:
Answer was 'Yes' at question '3 [PromotionJaNein]' (Have you completed a doctorate?)

❗ Choose one of the following answers
Please choose **only one** of the following:

- ◯ MD
- ◯ PhD
- ◯ Other

In which year (JJJJ) did you complete your doctorate? *

Only answer this question if the following conditions are met:
Answer was 'Yes' at question '3 [PromotionJaNein]' (Have you completed a doctorate?)

❗ Only numbers may be entered in this field.
Please write your answer here:

In which year did you receive your license to practice medicine? *

❗ Comment only when you choose an answer.
Please choose all that apply and provide a comment:

☐ Year:

☐ Not applicable

Which specialist training do you strive for or have you completed? *

❗ Choose one of the following answers
Please choose **only one** of the following:

○ Radiology

- ◯
- ◯ General medicine (family doctor)
- ◯ Anesthesiology
- ◯ Anatomy
- ◯ Occupational medicine (industrial medicine)
- ◯ Ophthalmology
- ◯ Biochemistry
- ◯ Surgery
- ◯ Gynecology and Obstetrics (Gynecology)
- ◯ Otolaryngology
- ◯ Skin and venereal diseases
- ◯ Human Genetics
- ◯ Hygiene and environmental medicine
- ◯ Internal Medicine
- ◯ Child and youth medicine
- ◯ Child and youth psychiatry and psychotherapy
- ◯ Laboratory Medicine
- ◯ Microbiology, virology and infection epidemiology
- ◯ Oral and maxillofacial surgery
- ◯ Neurosurgery
- ◯ Neurology
- ◯ Nuclear Medicine
- ◯ Public Health
- ◯ Pathology
- ◯ Pharmacology
- ◯ Phoniatrics and Pedaudiology
- ◯ Physical and rehabilitative medicine

- Physiology
- Psychiatry and Psychotherapy
- Psychosomatic medicine and psychotherapy
- Forensic Medicine
- Radiotherapy
- Transfusion Medicine
- Urology
- Other

What area of radiology interests you most? *

Only answer this question if the following conditions are met:
Answer was 'Radiology' at question '7 [FacharztAbgeschlZiel]' (Which specialist training do you strive for or have you completed?)

Check all that apply
Please choose **all** that apply:

- Nuclear Medicine
- Pediatric Radiology
- Interventional Radiology
- Neuroradiology
- Diagnostic Radiology
- No information
- Other:

When did you start and complete your specialist training?

Please enter month and year.

	Month(MM)	Year (JJJJ)
**Started**		
**Completed**		
**Expected completion**		

Did your specialist training take longer than planned or is it already foreseeable that your specialist training will take longer? *

Choose one of the following answers
Please choose **only one** of the following:

- Yes
- No
- No information

What were the reasons for extending your specialist training?

Multiple answers are possible

*

Only answer this question if the following conditions are met:
Answer was 'Yes' at question '10 [FAVerlaengerung]' (Did your specialist training take longer than planned or is it already foreseeable that your specialist training will take longer?)

❗ Check all that apply
Please choose **all** that apply:

- ☐ One or more job changes during the residency
- ☐ Part-time work during the specialist training
- ☐ Parental leave during specialist training
- ☐ Unavailability of rotation positions
- ☐ Illness
- ☐ Research activity
- ☐ Stay abroad
- ☐ Other:

II. Current Professional Situation

Which professional position do you currently hold? *

❗ Choose one of the following answers
Please choose **only one** of the following:

- ◯ Resident doctor
- ◯ Board certified / Specialist
- ◯ Senior physician / Consultant
- ◯ Leading senior physician
- ◯ Employed doctor in ambulant area
- ◯ Self-employed (own practice or participation in practice)
- ◯ Other

In your current position, to how many other physicians are you authorized to give instructions? *

❗ Choose one of the following answers
Please choose **only one** of the following:

- ◯ No authority to issue instructions
- ◯ 1-2 employees
- ◯ 3-9 employees
- ◯ 10 and more employees
- ◯ No information

Since when have you been working for your current employer?

Please enter month and year (MM/JJJJ).

*

❗ Only numbers may be entered in these fields.
Please write your answer(s) here:

month (MM)

year (JJJJ)

What is the term of your current employment contract? *

❗ Choose one of the following answers
Please choose **only one** of the following:

- ◯ Less than 1 year
- ◯ 1 year to 3 years
- ◯ More than 3 years
- ◯ Unlimited
- ◯ No information
- ◯ Other

Do you work full-time or part-time? *

Choose one of the following answers

Please choose **only one** of the following:

- ◯ Full time
- ◯ Part time
- ◯ No information
- ◯ Other

How many hours per week is your regular working time (excluding overtime)? *

Only numbers may be entered in these fields.

Please write your answer(s) here:

h/week

How many overtime hours do you work on average per week? How much overtime is paid or unpaid? *

❗ Only numbers may be entered in these fields.

Please write your answer(s) here:

Paid h/week

Unpaid h/week

On average, how often per month do you provide on-call and presence services? How many hours are paid or unpaid?

❗ Only numbers may be entered in these fields.

	**Paid**	**Unpaid**
**On-call service (number of hours per month):**		
**Presence services (number of hours per month):**		

What is your average gross monthly income including bonuses? *

Only answer this question if the following conditions are met:
Answer was 'Full time' at question '16 [VollVsTeilzeit]' (Do you work full-time or part-time?)

Choose one of the following answers
Please choose **only one** of the following:

- < 5.000 €
- > 5.000 € bis 7.000 €
- > 7.000 € bis 8.500 €
- > 8.500 bis 10.000 €
- > 10.000 €
- Other

What is your average gross monthly income including bonuses? *

Only answer this question if the following conditions are met:
Answer was 'Part time' *or* 'Other' *or* 'No information' at question '16 [VollVsTeilzeit]' (Do you work full-time or part-time?)

❶ Choose one of the following answers
Please choose **only one** of the following:

- ◯ < 3.000 €
- ◯ > 3.000 € bis 4.000 €
- ◯ > 4.000 € bis 5.000 €
- ◯ > 5.000 € bis 7.000 €
- ◯ > 7.000 € bis 8.500 €
- ◯ > 8.500 bis 10.000 €
- ◯ > 10.000 €
- ◯ Other

[ ]

On average, how much time (hours/week) do you spend on your research in your free time?

❶ Only integer values may be entered in these fields.
Please write your answer(s) here:

hours / week

[ ]

Do you work in your main job at a university hospital? *

❗ Choose one of the following answers

Please choose **only one** of the following:

- ◯ Yes
- ◯ No
- ◯ No information
- ◯ Other

[ ]

How many scientific papers have you already published? *

❗ Only numbers may be entered in these fields.

Please write your answer(s) here:

Total

[ ]

(of total) as first author

[ ]

(of total) last named author

[ ]

Have you already raised third-party funds? *

❗ Choose one of the following answers
Please choose **only one** of the following:

- Yes
- No
- No information
- Other

What is the approximate total amount of approved third-party funds?

Only answer this question if the following conditions are met:
Answer was 'Yes' at question '25 [Drittmittel]' (Have you already raised third-party funds?)

❗ Only integer values may be entered in these fields.
Please write your answer(s) here:

in Euro

Are you purely scientifically active? *

❗ Choose one of the following answers
Please choose **only one** of the following:

- ◯ Yes
- ◯ No
- ◯ No information
- ◯ Other

III. Job satisfaction and Personality Traits

What were your main expectations of your current job? *

Please choose the appropriate response for each item:

	very important	important	not so important	not important	not applicable
**Qualified training in health care**	◯	◯	◯	◯	◯
**Possibility for research activities**	◯	◯	◯	◯	◯
**Structured further education/compliance with further education times**	◯	◯	◯	◯	◯
**Opportunity for**					

**career development/prospects for promotion**	○	○	○	○	○
**Promotion of technical further education**	○	○	○	○	○
**Good income**	○	○	○	○	○
**Pleasure at work**	○	○	○	○	○
**Good working atmosphere**	○	○	○	○	○
**Family-friendly**	○	○	○	○	○
**Own creative leeway**	○	○	○	○	○
**Well plannable working hours & planning reliability**	○	○	○	○	○

To what extent have your main expectations of your current job been met? *

Please choose the appropriate response for each item:

	completely fulfilled	mainly fulfilled	barely fulfilled	not fulfilled at all	not applicable
**Qualified training in health care**	○	○	○	○	○

**Possibility for research activities**	◯	◯	◯	◯	◯
**Structured further education/compliance with further education times**	◯	◯	◯	◯	◯
**Opportunity for career development/prospects for promotion**	◯	◯	◯	◯	◯
**Promotion of technical further education**	◯	◯	◯	◯	◯
**Good income**	◯	◯	◯	◯	◯
**Pleasure at work**	◯	◯	◯	◯	◯
**Good working atmosphere**	◯	◯	◯	◯	◯
**Family-friendly**	◯	◯	◯	◯	◯
**Own creative leeway**	◯	◯	◯	◯	◯
**Well plannable working hours & planning reliability**	◯	◯	◯	◯	◯

Please answer some questions about your physical and mental well-being.

How often...

*

Please choose the appropriate response for each item:

	never	rarely	sometimes	often	always	no informatio
**... are you physically exhausted?**	○	○	○	○	○	○
**... are you emotionally exhausted?**	○	○	○	○	○	○
**... do you come to work although you feel sick and unwell?**	○	○	○	○	○	○
**... do you have the feeling of doing a meaningful job?**	○	○	○	○	○	○
**... do you have problems to switch off after work?**	○	○	○	○	○	○
**... do you feel**	○	○	○	○	○	○

**burdened by problematic decisions in patient care?**							

Here are a number of personality traits that may or may not apply to you. Please mark for each statement to what extent you agree or disagree with that statement. You should rate the extent to which the pair of traits applies to you, even if one characteristic applies more strongly than the other.

I see myself as:

*

Please choose the appropriate response for each item:

	Disagree strongly	Disagree moderately	Disagree a little	Neither agree nor disagree	Agree a little	Agree moderately	Agree strongly
**extraverted, enthusiastic**	○	○	○	○	○	○	○
**critical, quarrelsome**	○	○	○	○	○	○	○
**dependable, self-disciplined**	○	○	○	○	○	○	○
**anxious, easily upset**	○	○	○	○	○	○	○

**open to new experiences, complex**	◯	◯	◯	◯	◯	◯	◯
**reserved, quiet**	◯	◯	◯	◯	◯	◯	◯
**sympathetic, warm**	◯	◯	◯	◯	◯	◯	◯
**disorganized, careless**	◯	◯	◯	◯	◯	◯	◯
**calm, emotionally stable**	◯	◯	◯	◯	◯	◯	◯
**conventional, uncreative**	◯	◯	◯	◯	◯	◯	◯

To what extent do you feel supported in your professional activity by the following groups of people?

*

Please choose the appropriate response for each item:

	No support	Moderate support	A lot of support	Full support	Not applicable
**Supervisors**	○	○	○	○	○
**Colleagues**	○	○	○	○	○
**Employees**	○	○	○	○	○
**Spouse or life partner**	○	○	○	○	○
**Family**	○	○	○	○	○
**Friends and acquaintances**	○	○	○	○	○

How satisfied are/have you been with the following aspects of promoting your clinical knowledge/skills and (clinical) research?

*

Please choose the appropriate response for each item:

	Very satisfied	Rather satisfied	Rather dissatisfied	Very dissatisfied	Not applicable

... with the offer of employee interviews for career development	◯	◯	◯	◯	◯
... with the possibilities for clinical training	◯	◯	◯	◯	◯
... with the exemption for clinical research	◯	◯	◯	◯	◯
... with the possibility to organize congresses visits	◯	◯	◯	◯	◯
... with the integration in research projects	◯	◯	◯	◯	◯
... with the possibility to develop own projects and to apply for external funding	◯	◯	◯	◯	◯
... with the offer of supervision	◯	◯	◯	◯	◯
... with support in dealing with critical events	◯	◯	◯	◯	◯

Do you have or had a mentor*? *

❶ Choose one of the following answers
Please choose **only one** of the following:

- ◯ Yes
- ◯ No
- ◯ No information
- ◯ Other

[ ]

What gender is or was your mentor? *

Only answer this question if the following conditions are met:
Answer was 'Yes' at question '34 [MentorJaNein]' (Do you have or had a mentor*?)

❶ Choose one of the following answers
Please choose **only one** of the following:

- ◯ Female
- ◯ Male
- ◯ Divers
- ◯ No information
- ◯ Other

[ ]

Have you ever been bullied, harassed, or discriminated against in a professional situation? *

❗ Choose one of the following answers

Please choose **only one** of the following:

- ◯ No
- ◯ Yes
- ◯ No information
- ◯ Other

In what ways have you been bullied, harassed, or discriminated against?

Only answer this question if the following conditions are met:
Answer was 'Yes' at question '36 [MobbingJaNein]' (Have you ever been bullied, harassed, or discriminated against in a professional situation?)

Please write your answer here:

By whom were you bullied or harassed? *

Only answer this question if the following conditions are met:
Answer was 'Yes' *or* 'Other' at question '36 [MobbingJaNein]' (Have you ever been bullied, harassed, or discriminated against in a professional situation?)

Check all that apply
Please choose **all** that apply:

- [ ] by colleagues
- [ ] by superiors
- [ ] by patients
- [ ] no information
- [ ] Other:

Have you ever been sexually harassed? (e.g. verbal expressions, gestures, e-mails with unwanted content, unwanted physical contact, offers of advantages for sexual offers etc.)

*

Choose one of the following answers
Please choose **only one** of the following:

- No
- Yes
- No information
- Other

Who have you been sexually harassed by? *

Only answer this question if the following conditions are met:
Answer was 'Yes' *or* 'Other' at question '39 [SexuelleBelJaNein]' (Have you ever been sexually harassed? (e.g. verbal expressions, gestures, e-mails with unwanted content, unwanted physical contact, offers of advantages for sexual offers etc.))

❗ Check all that apply
Please choose **all** that apply:

- ☐ by colleagues
- ☐ by superiors
- ☐ by patients
- ☐ no information
- ☐ Other: ____________________

In what way were you sexually harassed?

Only answer this question if the following conditions are met:
Answer was 'Other' *or* 'Yes' at question '39 [SexuelleBelJaNein]' (Have you ever been sexually harassed? (e.g. verbal expressions, gestures, e-mails with unwanted content, unwanted physical contact, offers of advantages for sexual offers etc.))

❗ Check all that apply
Please choose **all** that apply:

- ☐ General derogatory or obscene sayings / jokes (not related to you personally)
- ☐ Insinuating or sexual references or derogatory or obscene remarks about you personally, your appearance or your clothing
- ☐ Unwanted letters, e-mails, phone calls etc. with derogatory or obscene jokes, sayings, pictures
- ☐ Whistling / staring
- ☐ Obscene gestures / signs
- ☐ Unwanted stories with sexual content
- ☐ Unsolicited invitations with sexual intent
- ☐ Pornographic material
- ☐ Unwanted body contact / seemingly random touches
- ☐ Unwanted fondling / kissing
- ☐ Sexual extortion / promise of advantages for sexual favors or threat of disadvantages in case no sexual favors are shown
- ☐ Sexual assault / rape
- ☐ Other:

Do you believe that your gender has had an impact on your career to date? *

Choose one of the following answers
Please choose **only one** of the following:

- Yes
- No
- No information

To what extent do you suspect that your gender has had an influence on your career to date?

Only answer this question if the following conditions are met:
Answer was 'Yes' at question '42 [EinflussGeschlecht]' (Do you believe that your gender has had an impact on your career to date?)

Please write your answer here:

IV. Career Aims

Do you strive for further specialization in research and teaching in order to obtain a professorship respectively do you already have a professorship?

*

❶ Choose one of the following answers

Please choose **only one** of the following:

- ◯ Yes, I already have an associate professorship.
- ◯ Yes, I have already started to specialize further in order to obtain an associate professorship.
- ◯ Yes, I am planning to begin my further specialization in order to obtain an associate professorship.
- ◯ I had the intention to get an associate professorship, but I rejected this plan.
- ◯ I do not know yet exactly.
- ◯ No, I do not intend to specialize further in order to obtain an associate professorship.
- ◯ No information

In which year did you achieve your associate professorship (YYYY) ? *

Only answer this question if the following conditions are met:
Answer was 'Yes, I already have an associate professorship.' at question '44 [Habilitation]' (Do you strive for further specialization in research and teaching in order to obtain a professorship respectively do you already have a professorship?)

❗ Only numbers may be entered in this field.
Please write your answer here:

Why do you strive for an associate professorship respectively why have you already achieved an associate professorship?

Please indicate the most important reasons. (multiple answers possible)

*

Only answer this question if the following conditions are met:
Answer was 'Yes, I already have an associate professorship.' *or* 'Yes, I have already started to specialize further in order to obtain an associate professorship.' *or* 'Yes, I am planning to begin my further specialization in order to obtain an associate professorship.' at question '44 [Habilitation]' (Do you strive for further specialization in research and teaching in order to obtain a professorship respectively do you already have a professorship?)

❗ Check all that apply
Please choose **all** that apply:

- ☐ To be able to apply for a professorship.
- ☐ To be able to apply for a position as chief physician.
- ☐ Out of interest in research and science.
- ☐ To have better opportunities for advancement outside the university.
- ☐ Out of interest in teaching.
- ☐ To get more development opportunities.
- ☐ To achieve greater independence.
- ☐ Other:

____

Why don't you strive for an associate professorship (anymore)?

Please indicate the most important reasons. (multiple answers possible)

*

Only answer this question if the following conditions are met:
Answer was 'I had the intention to get an associate professorship, but I rejected this plan.' *or* 'No, I do not intend to specialize further in order to obtain an associate professorship.' at question '44 [Habilitation]' (Do you strive for further specialization in research and teaching in order to obtain a professorship respectively do you already have a professorship?)

❗ Check all that apply
Please choose **all** that apply:

- ☐ Too much effort compared to the result.
- ☐ Not relevant for my professional plans.
- ☐ No exemption or subsidy.
- ☐ Not interested.
- ☐ Lack of compatibility with family.
- ☐ Other:

In your opinion, how helpful would the following offers and measures be to increase the attractiveness of management positions in university medicine? *

Please choose the appropriate response for each item:

	Decisive	Very helpful	Moderately helpful	Not very helpful	Not helpful at all
Flatter hierarchies	◯	◯	◯	◯	◯
Improved financial resources	◯	◯	◯	◯	◯
Offers for sharing management tasks ("top sharing")	◯	◯	◯	◯	◯
Dual-career offers for the life partner	◯	◯	◯	◯	◯
Gender balance at management level	◯	◯	◯	◯	◯
Measures to enhance the reputation of research and teaching in society	◯	◯	◯	◯	◯
Measures to make working hours more flexible (e.g. working time accounts)	◯	◯	◯	◯	◯
Offers for a better compatibility of work and family	◯	◯	◯	◯	◯

In which field of occupation would you like to work later? *

❶ Check all that apply

Please choose **all** that apply:

- ☐ As a self-employed physician in private practice in the outpatient sector
- ☐ As an employed physician in the outpatient sector
- ☐ As a physician in a university hospital
- ☐ As a physician in a hospital
- ☐ Outside of health care in the free economy
- ☐ Outside of health care in the public sector (e.g. medical service, public health department, non-university research
- ☐ As a physician in research
- ☐ Other:

All in all: What would you like to see improved career advancement in radiology or what would need to be improved?

(if necessary also other remarks)

*

Please write your answer here:

What is your academic position at the university? *

Choose one of the following answers
Please choose **only one** of the following:

- Full Professorship / Professor
- Associate Professor / Senior Lecturer
- Associate Professor / Adjunct Professor
- Endowed Professorship
- Assistent Professor / Lecturer
- Phd / MD / Teaching Assistent
- No information
- Other

V. Information about your Person and your private Circumstances

What is your gender? *

❶ Choose one of the following answers
Please choose **only one** of the following:

- ◯ Female
- ◯ Male
- ◯ Divers
- ◯ No information
- ◯ Other

In which year were you born? *

❶ Only integer values may be entered in these fields.
Please write your answer(s) here:

Year (JJJJ)

In which country were you born? *

❶ Choose one of the following answers
Please choose **only one** of the following:

- ◯ Germany
- ◯ USA

- Egypt
- Equatorial Guinea
- Ethiopia
- Afghanistan
- Åland Islands
- Albanien
- Algeria
- American Samoa
- U.S. Virgin Islands
- Andorra
- Angola
- Anguilla
- Antarctica
- Antigua and Barbuda
- Argentina
- Armenia
- Aruba
- Azerbaijan
- Australia
- Bahamas
- Bahrain
- Bangladesh
- Barbados
- Belgium
- Belize
- Benin
- Bermuda

- Bhutan
- Bolivia
- Bosnia and Herzegovina
- Botswana
- Bouvet Island
- Brazil
- British Virgin Islands
- British Indian Ocean Territory
- Brunei
- Bulgaria
- Burkina Faso
- Burundi
- Chile
- China
- Cook Islands
- Costa Rica
- Denmark
- Democratic Republic of the Congo
- Germany
- Dominica
- Dominican Republic
- Djibouti
- Ecuador
- El Salvador
- Ivory Coast
- Eritrea
- Estonia

- Falkland Islands
- Faroe Islands
- Fiji
- Finland
- France
- French Guiana
- French Southern Territories
- French Polynesia
- Gabon
- Gambia
- Georgia
- Ghana
- Gibraltar
- Grenada
- Greece
- Greenland
- United Kingdom
- Guadeloupe
- Guam
- Guatemala
- Guernsey
- Guinea
- Guinea-Bissau
- Guyana
- Haiti
- Heard Island and McDonald Islands
- Honduras

- Hong Kong
- India
- Indonesia
- Iraq
- Iran
- Ireland
- Iceland
- Isle of Man
- Israel
- Italy
- Jamaica
- Japan
- Yemen
- Jersey
- Jordan
- Cayman Islands
- Cambodia
- Cameroon
- Canada
- Cape Verde
- Kazakhstan
- Qatar
- Kenya
- Kyrgyzstan
- Kiribati
- Cocos Islands
- Colombia

- Comoros
- Kosovo
- Croatia
- Cuba
- Kuwait
- Laos
- Lesotho
- Latvia
- Lebanon
- Liberia
- Libya
- Liechtenstein
- Lithuania
- Luxembourg
- Macao
- Madagascar
- Malawi
- Malaysia
- Maldives
- Mali
- Malta
- Morocco
- Marshall Islands
- Martinique
- Mauritania
- Mauritius
- Mayotte

- ◯ Macedonia
- ◯ Mexico
- ◯ Micronesia
- ◯ Moldova
- ◯ Monaco
- ◯ Mongolia
- ◯ Montenegro
- ◯ Montserrat
- ◯ Mozambique
- ◯ Myanmar
- ◯ Namibia
- ◯ Nauru
- ◯ Nepal
- ◯ New Caledonia
- ◯ New Zealand
- ◯ Nicaragua
- ◯ Netherlands Antilles
- ◯ Netherlands
- ◯ Niger
- ◯ Nigeria
- ◯ Niue
- ◯ North Korea
- ◯ Norfolk Island
- ◯ Northern Mariana Islands
- ◯ Norway
- ◯ Austria
- ◯ Oman

- East Timor
- Pakistan
- Palestinian Territory
- Palau
- Panama
- Papua New Guinea
- Paraguay
- Peru
- Philippines
- Pitcairn
- Poland
- Portugal
- Puerto Rico
- Republic of the Congo
- Reunion
- Rwanda
- Romania
- Russia
- Saint Helena
- Saint Kitts and Nevis
- Saint Martin
- Saint Pierre and Miquelon
- Saint Bartholemy
- Solomon Islands
- Zambia
- Samoa
- San Marino

- ○ Sao Tome and Principe
- ○ Saudi Arabia
- ○ Sweden
- ○ Switzerland
- ○ Senegal
- ○ Serbia
- ○ Serbia and Montenegro
- ○ Seychelles
- ○ Sierra Leone
- ○ Zimbabwe
- ○ Singapore
- ○ Slovakia
- ○ Slovenia
- ○ Somalia
- ○ Spain
- ○ Svalbard and Jan Mayen
- ○ Sri Lanka
- ○ Saint Lucia
- ○ Saint Vincent and the Grenadines
- ○ Sudan
- ○ South Africa
- ○ South Georgia and the South Sandwich Islands
- ○ South Korea
- ○ Suriname
- ○ Swaziland
- ○ Syria
- ○ Tajikistan

- Taiwan
- Tanzania
- Thailand
- Togo
- Tokelau
- Tonga
- Trinidad and Tobago
- Chad
- Czech Republic
- Turkey
- Tunisia
- Turkmenistan
- Turks and Caicos Islands
- Tuvalu
- Uganda
- Ukraine
- Hungary
- United States Minor Outlying Islands
- Uruguay
- USA
- Uzbekistan
- Vanuatu
- Vatican
- Venezuela
- United Arab Emirates
- Vietnam
- Wallis and Futuna

- Christmas Island
- Belarus
- Western Sahara
- Central African Republic
- Cyprus
- Other

Please indicate the country of your citizenship. *

Choose one of the following answers

Please choose **only one** of the following:

- Germany
- USA
- Egypt
- Equatorial Guinea
- Ethiopia
- Afghanistan
- Åland Islands
- Albanien
- Algeria
- American Samoa
- U.S. Virgin Islands
- Andorra
- Angola

- ○ Anguilla
- ○ Antarctica
- ○ Antigua and Barbuda
- ○ Argentina
- ○ Armenia
- ○ Aruba
- ○ Azerbaijan
- ○ Australia
- ○ Bahamas
- ○ Bahrain
- ○ Bangladesh
- ○ Barbados
- ○ Belgium
- ○ Belize
- ○ Benin
- ○ Bermuda
- ○ Bhutan
- ○ Bolivia
- ○ Bosnia and Herzegovina
- ○ Botswana
- ○ Bouvet Island
- ○ Brazil
- ○ British Virgin Islands
- ○ British Indian Ocean Territory
- ○ Brunei
- ○ Bulgaria
- ○ Burkina Faso

- ◯ Burundi
- ◯ Chile
- ◯ China
- ◯ Cook Islands
- ◯ Costa Rica
- ◯ Denmark
- ◯ Democratic Republic of the Congo
- ◯ Germany
- ◯ Dominica
- ◯ Dominican Republic
- ◯ Djibouti
- ◯ Ecuador
- ◯ El Salvador
- ◯ Ivory Coast
- ◯ Eritrea
- ◯ Estonia
- ◯ Falkland Islands
- ◯ Faroe Islands
- ◯ Fiji
- ◯ Finland
- ◯ France
- ◯ French Guiana
- ◯ French Southern Territories
- ◯ French Polynesia
- ◯ Gabon
- ◯ Gambia
- ◯ Georgia

- Ghana
- Gibraltar
- Grenada
- Greece
- Greenland
- United Kingdom
- Guadeloupe
- Guam
- Guatemala
- Guernsey
- Guinea
- Guinea-Bissau
- Guyana
- Haiti
- Heard Island and McDonald Islands
- Honduras
- Hong Kong
- India
- Indonesia
- Iraq
- Iran
- Ireland
- Iceland
- Isle of Man
- Israel
- Italy
- Jamaica

- Japan
- Yemen
- Jersey
- Jordan
- Cayman Islands
- Cambodia
- Cameroon
- Canada
- Cape Verde
- Kazakhstan
- Qatar
- Kenya
- Kyrgyzstan
- Kiribati
- Cocos Islands
- Colombia
- Comoros
- Kosovo
- Croatia
- Cuba
- Kuwait
- Laos
- Lesotho
- Latvia
- Lebanon
- Liberia
- Libya

- ◯ Liechtenstein
- ◯ Lithuania
- ◯ Luxembourg
- ◯ Macao
- ◯ Madagascar
- ◯ Malawi
- ◯ Malaysia
- ◯ Maldives
- ◯ Mali
- ◯ Malta
- ◯ Morocco
- ◯ Marshall Islands
- ◯ Martinique
- ◯ Mauritania
- ◯ Mauritius
- ◯ Mayotte
- ◯ Macedonia
- ◯ Mexico
- ◯ Micronesia
- ◯ Moldova
- ◯ Monaco
- ◯ Mongolia
- ◯ Montenegro
- ◯ Montserrat
- ◯ Mozambique
- ◯ Myanmar
- ◯ Namibia

- Nauru
- Nepal
- New Caledonia
- New Zealand
- Nicaragua
- Netherlands Antilles
- Netherlands
- Niger
- Nigeria
- Niue
- North Korea
- Norfolk Island
- Northern Mariana Islands
- Norway
- Austria
- Oman
- East Timor
- Pakistan
- Palestinian Territory
- Palau
- Panama
- Papua New Guinea
- Paraguay
- Peru
- Philippines
- Pitcairn
- Poland

- Portugal
- Puerto Rico
- Republic of the Congo
- Reunion
- Rwanda
- Romania
- Russia
- Saint Helena
- Saint Kitts and Nevis
- Saint Martin
- Saint Pierre and Miquelon
- Saint Bartholemy
- Solomon Islands
- Zambia
- Samoa
- San Marino
- Sao Tome and Principe
- Saudi Arabia
- Sweden
- Switzerland
- Senegal
- Serbia
- Serbia and Montenegro
- Seychelles
- Sierra Leone
- Zimbabwe
- Singapore

- ◯ Slovakia
- ◯ Slovenia
- ◯ Somalia
- ◯ Spain
- ◯ Svalbard and Jan Mayen
- ◯ Sri Lanka
- ◯ Saint Lucia
- ◯ Saint Vincent and the Grenadines
- ◯ Sudan
- ◯ South Africa
- ◯ South Georgia and the South Sandwich Islands
- ◯ South Korea
- ◯ Suriname
- ◯ Swaziland
- ◯ Syria
- ◯ Tajikistan
- ◯ Taiwan
- ◯ Tanzania
- ◯ Thailand
- ◯ Togo
- ◯ Tokelau
- ◯ Tonga
- ◯ Trinidad and Tobago
- ◯ Chad
- ◯ Czech Republic
- ◯ Turkey
- ◯ Tunisia

- Turkmenistan
- Turks and Caicos Islands
- Tuvalu
- Uganda
- Ukraine
- Hungary
- United States Minor Outlying Islands
- Uruguay
- USA
- Uzbekistan
- Vanuatu
- Vatican
- Venezuela
- United Arab Emirates
- Vietnam
- Wallis and Futuna
- Christmas Island
- Belarus
- Western Sahara
- Central African Republic
- Cyprus
- Other

In which country do you currently work? *

❗ Choose one of the following answers

Please choose **only one** of the following:

- ◯ Germany
- ◯ USA
- ◯ Egypt
- ◯ Equatorial Guinea
- ◯ Ethiopia
- ◯ Afghanistan
- ◯ Åland Islands
- ◯ Albanien
- ◯ Algeria
- ◯ American Samoa
- ◯ U.S. Virgin Islands
- ◯ Andorra
- ◯ Angola
- ◯ Anguilla
- ◯ Antarctica
- ◯ Antigua and Barbuda
- ◯ Argentina
- ◯ Armenia
- ◯ Aruba
- ◯ Azerbaijan
- ◯ Australia
- ◯ Bahamas

- ◯ Bahrain
- ◯ Bangladesh
- ◯ Barbados
- ◯ Belgium
- ◯ Belize
- ◯ Benin
- ◯ Bermuda
- ◯ Bhutan
- ◯ Bolivia
- ◯ Bosnia and Herzegovina
- ◯ Botswana
- ◯ Bouvet Island
- ◯ Brazil
- ◯ British Virgin Islands
- ◯ British Indian Ocean Territory
- ◯ Brunei
- ◯ Bulgaria
- ◯ Burkina Faso
- ◯ Burundi
- ◯ Chile
- ◯ China
- ◯ Cook Islands
- ◯ Costa Rica
- ◯ Denmark
- ◯ Democratic Republic of the Congo
- ◯ Germany
- ◯ Dominica

- Dominican Republic
- Djibouti
- Ecuador
- El Salvador
- Ivory Coast
- Eritrea
- Estonia
- Falkland Islands
- Faroe Islands
- Fiji
- Finland
- France
- French Guiana
- French Southern Territories
- French Polynesia
- Gabon
- Gambia
- Georgia
- Ghana
- Gibraltar
- Grenada
- Greece
- Greenland
- United Kingdom
- Guadeloupe
- Guam
- Guatemala

- Guernsey
- Guinea
- Guinea-Bissau
- Guyana
- Haiti
- Heard Island and McDonald Islands
- Honduras
- Hong Kong
- India
- Indonesia
- Iraq
- Iran
- Ireland
- Iceland
- Isle of Man
- Israel
- Italy
- Jamaica
- Japan
- Yemen
- Jersey
- Jordan
- Cayman Islands
- Cambodia
- Cameroon
- Canada
- Cape Verde

- ◯ Kazakhstan
- ◯ Qatar
- ◯ Kenya
- ◯ Kyrgyzstan
- ◯ Kiribati
- ◯ Cocos Islands
- ◯ Colombia
- ◯ Comoros
- ◯ Kosovo
- ◯ Croatia
- ◯ Cuba
- ◯ Kuwait
- ◯ Laos
- ◯ Lesotho
- ◯ Latvia
- ◯ Lebanon
- ◯ Liberia
- ◯ Libya
- ◯ Liechtenstein
- ◯ Lithuania
- ◯ Luxembourg
- ◯ Macao
- ◯ Madagascar
- ◯ Malawi
- ◯ Malaysia
- ◯ Maldives
- ◯ Mali

- Malta
- Morocco
- Marshall Islands
- Martinique
- Mauritania
- Mauritius
- Mayotte
- Macedonia
- Mexico
- Micronesia
- Moldova
- Monaco
- Mongolia
- Montenegro
- Montserrat
- Mozambique
- Myanmar
- Namibia
- Nauru
- Nepal
- New Caledonia
- New Zealand
- Nicaragua
- Netherlands Antilles
- Netherlands
- Niger
- Nigeria

- ◯ Niue
- ◯ North Korea
- ◯ Norfolk Island
- ◯ Northern Mariana Islands
- ◯ Norway
- ◯ Austria
- ◯ Oman
- ◯ East Timor
- ◯ Pakistan
- ◯ Palestinian Territory
- ◯ Palau
- ◯ Panama
- ◯ Papua New Guinea
- ◯ Paraguay
- ◯ Peru
- ◯ Philippines
- ◯ Pitcairn
- ◯ Poland
- ◯ Portugal
- ◯ Puerto Rico
- ◯ Republic of the Congo
- ◯ Reunion
- ◯ Rwanda
- ◯ Romania
- ◯ Russia
- ◯ Saint Helena
- ◯ Saint Kitts and Nevis

- ◯ Saint Martin
- ◯ Saint Pierre and Miquelon
- ◯ Saint Bartholemy
- ◯ Solomon Islands
- ◯ Zambia
- ◯ Samoa
- ◯ San Marino
- ◯ Sao Tome and Principe
- ◯ Saudi Arabia
- ◯ Sweden
- ◯ Switzerland
- ◯ Senegal
- ◯ Serbia
- ◯ Serbia and Montenegro
- ◯ Seychelles
- ◯ Sierra Leone
- ◯ Zimbabwe
- ◯ Singapore
- ◯ Slovakia
- ◯ Slovenia
- ◯ Somalia
- ◯ Spain
- ◯ Svalbard and Jan Mayen
- ◯ Sri Lanka
- ◯ Saint Lucia
- ◯ Saint Vincent and the Grenadines
- ◯ Sudan

- South Africa
- South Georgia and the South Sandwich Islands
- South Korea
- Suriname
- Swaziland
- Syria
- Tajikistan
- Taiwan
- Tanzania
- Thailand
- Togo
- Tokelau
- Tonga
- Trinidad and Tobago
- Chad
- Czech Republic
- Turkey
- Tunisia
- Turkmenistan
- Turks and Caicos Islands
- Tuvalu
- Uganda
- Ukraine
- Hungary
- United States Minor Outlying Islands
- Uruguay
- USA

- ◯ Uzbekistan
- ◯ Vanuatu
- ◯ Vatican
- ◯ Venezuela
- ◯ United Arab Emirates
- ◯ Vietnam
- ◯ Wallis and Futuna
- ◯ Christmas Island
- ◯ Belarus
- ◯ Western Sahara
- ◯ Central African Republic
- ◯ Cyprus
- ◯ Other

What is your marital status? *

❶ Choose one of the following answers
Please choose **only one** of the following:

- ◯ Married
- ◯ Registered partnership
- ◯ Living in a solid partnership
- ◯ Without a permanent partnership
- ◯ Divorced
- ◯ Widowed
- ◯ No information
- ◯ Other

Is your partner currently employed? *

Only answer this question if the following conditions are met:
Answer was 'Married' *or* 'Registered partnership' *or* 'Living in a solid partnership' at question '57 [Familienstand]' (What is your marital status?)

❶ Choose one of the following answers
Please choose **only one** of the following:

- ◯ Yes
- ◯ No

To what extent is your partner currently employed?

Please indicate the average working hours per week!

*

Only answer this question if the following conditions are met:
Answer was 'Yes' at question '58 [PartnerBeruf]' (Is your partner currently employed?)

Only numbers may be entered in these fields.
Please write your answer(s) here:

h/week

What is your partner's profession?

Please enter the job title as precisely as possible. (e.g. teacher, nurse, office clerk etc.)!

*

Only answer this question if the following conditions are met:
((Familienstand.NAOK (/admin/questions/sa/view/surveyid/267763/gid/31/qid/755) == "A1" or Familienstand.NAOK (/admin/questions/sa/view/surveyid/267763/gid/31/qid/755) == "A2" or Familienstand.NAOK (/admin/questions/sa/view/surveyid/267763/gid/31/qid/755) == "A3"))

Please write your answer here:

Do you have children? *

Choose one of the following answers
Please choose **only one** of the following:

- Yes
- No

How many children do you have?

Only answer this question if the following conditions are met:
Answer was 'Yes' at question '61 [KinderJaNein]' (Do you have children?)

Please write your answer(s) here:

child(ren):

How old are your children?

If under one year, please enter 0.

Only answer this question if the following conditions are met:
Answer was 'Yes' at question '61 [KinderJaNein]' (Do you have children?)

Please write your answer(s) here:

Age of the oldest or only child:

Age of the youngest child:

How is/were your child(ren) mainly looked after during the day?

Multiple answers possible

*

Only answer this question if the following conditions are met:
Answer was 'Yes' at question '61 [KinderJaNein]' (Do you have children?)

Check all that apply

Please choose **all** that apply:

- [ ] By myself
- [ ] By my partner
- [ ] By other family members
- [ ] By a day mother, au pair, friends, acquaintances or similar
- [ ] By a public institution
- [ ] No Information
- [ ] Other:

Have you or your partner interrupted your own professional activity so far due to childcare?

Maternity leave does not count as an interruption! Multiple answers possible. Parental leave on the other hand does count as an interruption.

*

Only answer this question if the following conditions are met:
Answer was 'Yes' at question '61 [KinderJaNein]' (Do you have children?)

❗ Comment only when you choose an answer.
Please choose all that apply and provide a comment:

☐ Yes, I have interrupted my professional activity "X times" so far. Please enter the number:

☐ Yes, my spouse / partner has interrupted his or her professional activity "X times" so far. Please enter the number:

☐ Yes, my partner has suspended his or her work until further notice in favor of childcare.

☐ No, I have not or we have not interrupted our professional activities so far.

☐ No information

Other:

Over what period of time did the interruptions run in total?

Please enter the period in months.

*

Only answer this question if the following conditions are met:
-------- Scenario 1 --------
Answer was at question '65 [KinderBerufBreak]' (Have you or your partner interrupted your own professional activity so far due to childcare? Maternity leave does not count as an interruption! Multiple answers possible. Parental leave on the other hand does count as an interruption.)
-------- or Scenario 2 --------
Answer was at question '65 [KinderBerufBreak]' (Have you or your partner interrupted your own professional activity so far due to childcare? Maternity leave does not count as an interruption! Multiple answers possible. Parental leave on the other hand does count as an interruption.)
-------- or Scenario 3 --------
Answer was at question '65 [KinderBerufBreak]' (Have you or your partner interrupted your own professional activity so far due to childcare? Maternity leave does not count as an interruption! Multiple answers possible. Parental leave on the other hand does count as an interruption.)

Please write your answer(s) here:

Own interruption. Duration in months:

Interruption of the partner. Duration in months:

You have successfully completed the study. Thank you very much for your participation.

If you have further questions, please contact i.molwitz@uke.de

03-31-2022 – 14:55

Submit your survey.
Thank you for completing this survey.
